# Case Report: Vertical muscle-sparing latissimus dorsi flap in the reconstruction of chronic radiation-induced chest wall ulcers after breast cancer surgery: a case series

**DOI:** 10.3389/fsurg.2024.1397233

**Published:** 2024-07-26

**Authors:** Sung Joon Han, Junghee Kim, Sunje Kim, Yooseok Ha

**Affiliations:** ^1^ Department of Thoracic and Cardiovascular Surgery, Chungnam National University Hospital, Daejeon, Republic of Korea; ^2^ Department of Plastic and Reconstructive Surgery, Chungnam National University Hospital, Daejeon, Republic of Korea

**Keywords:** breast cancer, radiation therapy, chronic ulcers, latissimus dorsi flap, flap surgery, reconstruction

## Abstract

**Introduction:**

Radiation therapy, a standard postoperative treatment for breast cancer, can lead to chronic ulcers owing to compromised tissue healing. Accordingly, flap surgery using healthy tissues is essential for aesthetic and functional recovery. Although various flap techniques exist, each has its own drawbacks. This study introduces the vertical muscle-sparing latissimus dorsi flap as a superior alternative due to its comparative operative efficiency and tissue preservation.

**Case report:**

Two female patients aged 60 and 59 years with histories of breast cancer in their left breast treated with radiotherapy presented with chronic ulcers. The first patient had a 4 × 5 cm defect infiltrating the pleural space, while the second had a 15 × 9 cm defect after thoracic surgery for a bronchopleural fistula. In both cases, debridement was followed by reconstruction using a vertical muscle-sparing latissimus dorsi flap, thereby avoiding the need to change the patient's position and repeated draping during surgery. Both patients showed good postoperative recovery without significant complications.

**Results:**

The vertical muscle-sparing latissimus dorsi flap resulted in better adhesions and functional outcomes due to shorter surgical duration.

**Conclusion:**

The vertical muscle-sparing latissimus dorsi flap is an effective and efficient method for reconstructing radiation-induced chest wall ulcers in patients with breast cancer. Its application in the presented cases highlights its potential as a preferred option in similar clinical scenarios.

## 1 Introduction

Breast cancer is a leading cause of morbidity and mortality among women worldwide, and surgical resection remains the cornerstone of its treatment (1). Moreover, postoperative radiation therapy, a vital component of breast cancer management, is not without its complications (2). One such notable complication, the development of chronic ulcers in the chest wall, poses a significant challenge to both the patient and the surgeon (3).

Chronic ulcers resulting from radiation therapy often represent a complex clinical problem as radiation compromises the integrity and regenerative capacity of the skin and underlying tissues, rendering them susceptible to ulceration. The management of these ulcers is not straightforward as they frequently require surgical intervention to achieve both aesthetic and functional recovery.

Several surgical methods, including the free flap, pedicled transverse rectus abdominis myocutaneous (TRAM) flap (4), and conventional latissimus dorsi (LD) flap (5), are available for the reconstruction of chronic ulcers in the chest following breast cancer surgery. Each of which has specific advantages and disadvantages. The free flap, while versatile, may increase surgical time and complexity, particularly in patients with compromised recipient vessels due to prior radiation treatment (3). The pedicled TRAM flap offers a substantial amount of tissue for coverage but necessitates the sacrifice of the rectus abdominis muscle. The conventional LD flap, commonly employed for chest wall reconstruction, often requires repositioning of the patient during surgery from lateral to supine, which complicates the surgical process (6).

In response to these challenges, the vertical muscle-sparing LD flap emerges as a promising alternative (7). This technique boasts several advantages over traditional methods, including reduced operation time, minimal need for intraoperative positional changes, and preservation of muscle integrity (8). These benefits make the vertical muscle-sparing LD flap a valuable option for reconstructing chronic radiation-induced ulcers in the chest wall following breast cancer surgery. In this study, we describe our experience with a vertical muscle-sparing LD flap in the reconstruction of the chest wall in two patients with chronic ulcers after breast cancer therapy, highlighting the practical and clinical advantages of this innovative approach.

## 2 Case description

### 2.1 Patient 1

**Figure 1:**
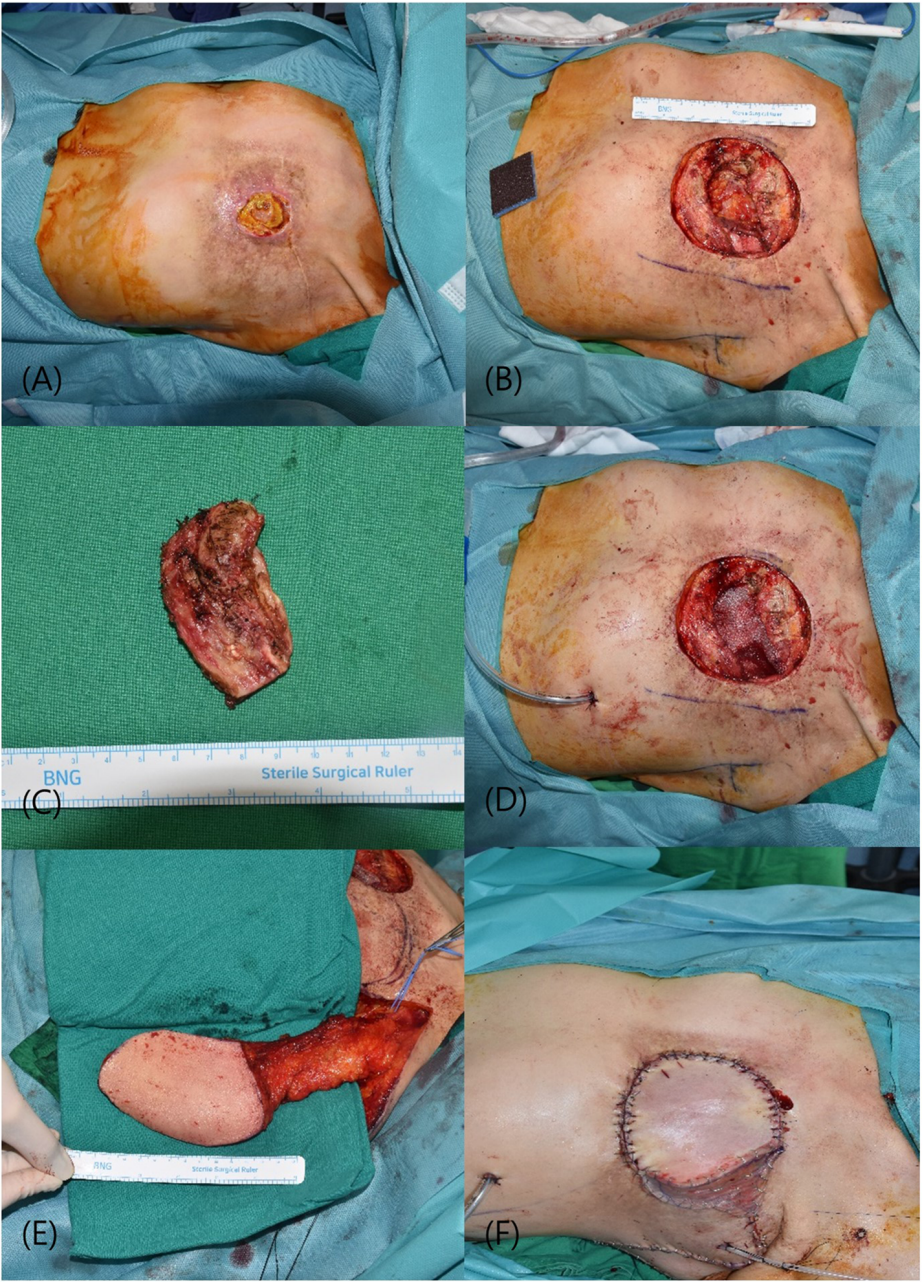
(**A**) initial presentation of a 4 × 5 cm defect in the chest wall. (**B**) Post-excision of necrotic skin and soft tissue, revealing the underlying structural deficit. (**C**) The resected rib segment exhibiting osteonecrosis. (**D**) A 10 × 8 cm defect in the chest wall, now stabilized with a synthetic mesh, providing a scaffold for tissue integration. (**E**) Preparation of a vertically oriented LD myocutaneous flap prior to its transfer. (**F**) Completion of the reconstruction with a split-thickness skin graft applied to the raw surfaces. LD, latissimus dorsi.

A 60-year-old female, with a history of left breast cancer that was diagnosed 26 years prior, presented with complications following radiotherapy after mastectomy. The patient experienced radiotherapy-induced dermatitis requiring continuous disinfection. Upon presentation to our hospital, she exhibited a 4 × 5 cm chest wall defect. Preoperative chest CT revealed that the defect extended to the pleural space at the rib level. During surgery, friable skin and soft tissues weakened by radiotherapy were excised, including the ribs affected by osteonecrosis. The resulting defect, measuring 10 × 8 cm, was covered with a mesh. Reconstruction was performed using the muscle-sparing vertical LD method. In this approach, the anterior margin of the LD muscle was identified and the descending branch of the thoracodorsal vessel was carefully dissected. The horizontal branch of the thoracodorsal nerve was preserved to maintain its function. The skin flap was designed, elevated accordingly, and placed over the defect via subcutaneous tunneling. The remaining raw surfaces were covered using split-thickness skin grafts (STSG). The patient began ward ambulation 2 days after surgery and was discharged on the 8th day with no significant wound issues or donor site complications. Follow-up over two months postoperatively revealed no additional complications (Figure 1).

### 2.2 Patient 2

**Figure 2:**
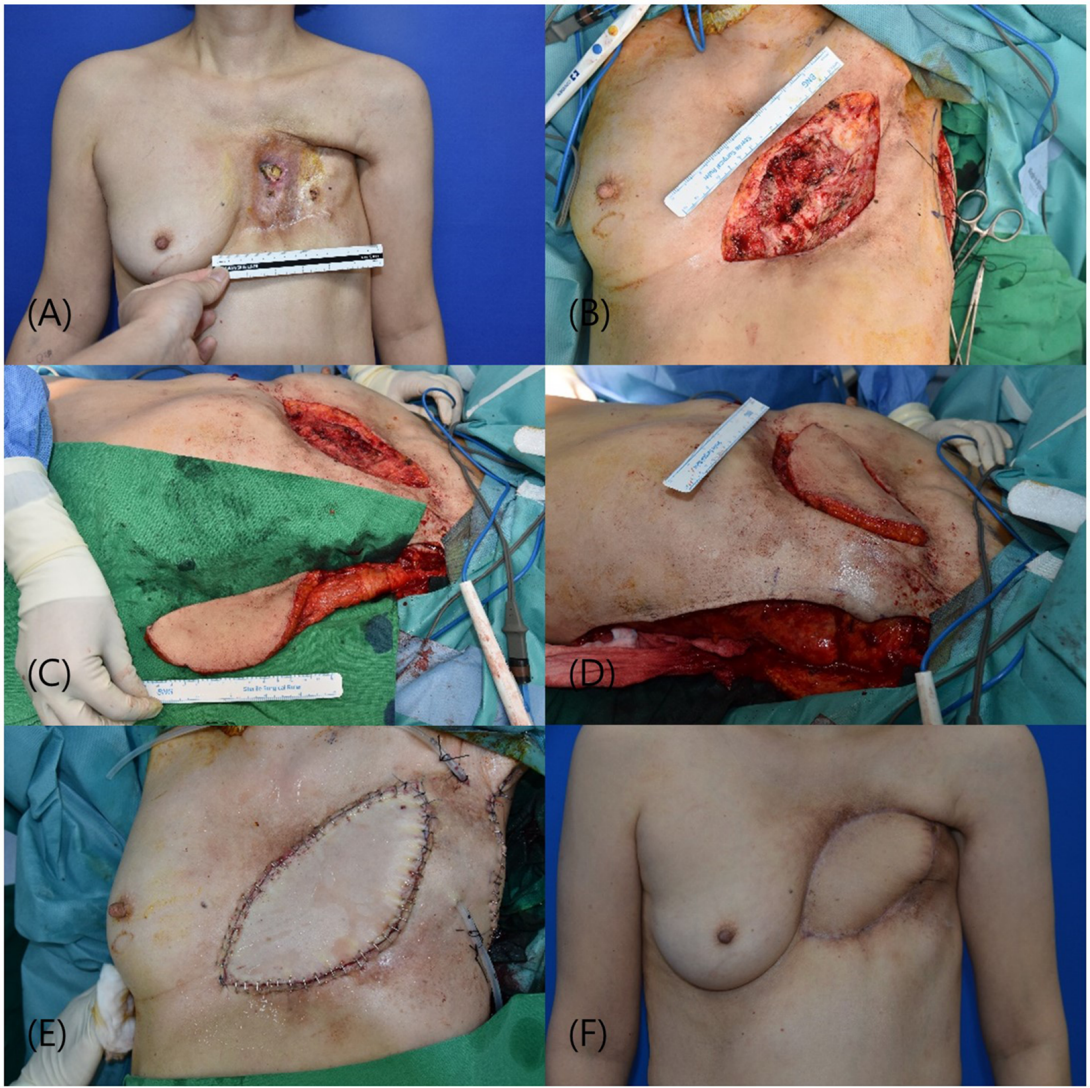
(**A**) presentation of a 15 × 9 cm defect in the chest wall, necessitating complex surgical management. (**B**) Following excision of the compromised skin and soft tissue, post successful closure of a bronchopleural fistula. (**C**) Harvesting of a vertically oriented LD myocutaneous flap in preparation for translocation to the chest wall defect. (**D**) Subsequent insetting and integration of the flap, providing robust coverage. (**E**) Immediate postoperative appearance following reconstructive surgery. (**F**) The patient's anterior view four weeks post-surgery, showing satisfactory healing and contour restoration without complications. LD, latissimus dorsi.

The patient was a 59-year-old female with a history of left breast cancer that was initially diagnosed 26 years previously. Following neo-adjuvant chemotherapy and a modified radical mastectomy, the patient underwent radiation therapy. The patient subsequently developed a bronchopleural fistula requiring thoracic surgery. The patient presented with arterial bleeding due to a chronic ulcer. Similar to the first case, combined thoracic surgery was performed, with our department conducting skin excision and the thoracic surgery team performing bony thoracic bed curettage. Following the initiation of a skin incision and exposure of the chest wall, including the ribs, the surgical procedure was commenced with the thoracic surgery department. Initially, access to the thoracic cavity was gained through the intercostal space, and the presence of the bronchopleural fistula (BPF) was visually confirmed. In the early stages of fistula appearance, immediate measures involve surgical repair of the BPF and thoracotomic debridement of the necrotic tissue. The bronchial stump was meticulously isolated, dissected, and sutured using non-absorbable 3-0 braided threads. The final defect size was 15 × 9 cm. Reconstruction using the muscle-sparing vertical LD flap method, as employed in the first patient, was performed. Most of the donor defects were primarily closed; however, a section could not be completely closed, requiring coverage by a STSG. The patient began ward ambulation 3 days postoperatively, and the bolster at the donor site was removed on the 5th day. The patient was discharged after a six-day hospital stay without any specific complications at either the operative or donor sites. Follow-up at four months postoperatively revealed no additional complications (Figure 2).

### 2.3 Operative technique

**Figure 3:**
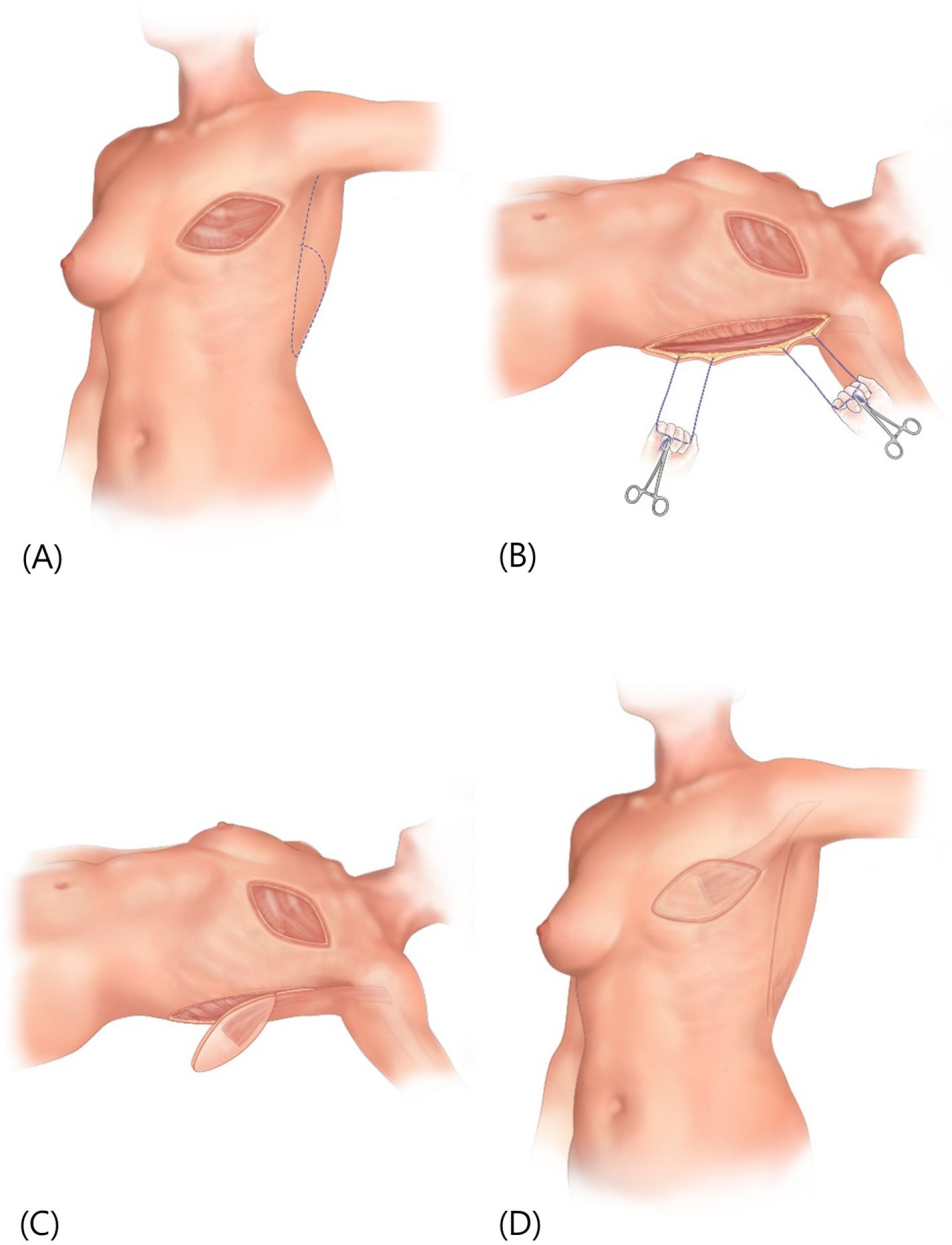
(**A**) preoperative markings on the patient's chest represent the boundaries of the surgical area, with a dotted line indicating the potential flap design for reconstruction. (**B**) Retractors are utilized to pull the tissue aside, revealing the lateral border of the LD muscle. A dissection is made beneath this border. (**C**) The creation of a muscle pedicle and the design of a skin flap positioned over it. The flap has been elevated in preparation for transfer. (**D**) The initial defect covered by the transferred flap, and the donor site area closed primarily. LD, latissimus dorsi.

The patient is positioned in supine with the arm of the affected side abducted at 90° and a cushion is placed behind the scapula to expose the lateral border of the LD. Using the pinch test, the lateral border of the LD is palpated and an incision is made 2 cm anterior to the anticipated border. After identifying the LD border, dissection is performed under the LD surface to confirm the course of the descending branch of the thoracodorsal vessel. Including the thoracodorsal vessel, a muscle flap approximately 5–10 cm from the LD margin is created as the pedicle. To enhance perfusion, the skin flap is designed to be placed over the muscle pedicle, incorporating as much of the muscle pedicle as possible, before making the skin flap incision. However, if a long arc of rotation is required, such as in the medial portion of the breast, only approximately half of the proximal part of the LD muscle is attached to the skin flap, allowing coverage of relatively distant defects. The flap is transferred to the defect via subcutaneous tunneling, and the donor site is closed primarily, or if not possible, with an STSG (Figure 3).

## 3 Discussion

Radiotherapy, a crucial adjunct in the management of breast cancer, presents a paradoxical challenge. It significantly reduces local cancer recurrence and improves survival rates, but also poses a risk of severe complications (9), such as radiation-induced ulcers in normal tissues, which initiate the formation of non-healing ulcers and soft tissue necrosis. As highlighted in prior studies, moderate-to-severe skin reactions occur in a substantial proportion of patients (up to 85%) undergoing radiotherapy (10). Chronic radiation-induced ulcers in the chest wall exhibit both progressive and irreversible characteristics. These ulcers represent a formidable challenge owing to their resistance to self-healing, which is attributed to compromised blood supply, tissue fibrosis, and diminished cellular regenerative capacity following radiation exposure. Additionally, chronic radiation-induced ulcers in the chest wall exhibit diverse clinical manifestations, encompassing variations in ulcer size, location, depth, and basal conditions, which influence decisions regarding treatment or reconstruction methods in clinical practice. Nevertheless, the optimization of treatment selection for these ulcers remains an ongoing challenge.

The complexity of these ulcers necessitates a tailored approach that encompasses debridement of an appropriate breadth and depth. This includes not only the removal of the ulcerated tissue but also any radiologically evident changes in the surrounding skin tissue. In patients presenting with radiation-induced osteomyelitis, excision of the affected bone is also crucial (11). Furthermore, preserving the integrity of deeper structures, such as the pleural cavity, is essential unless direct involvement mandates intervention. Therefore, meticulous surgical intervention is paramount for managing these complications.

Following debridement, various reconstructive strategies can be employed, ranging from conservative therapy and primary closure to more complex procedures, such as local or free flaps. However, each technique has limitations. For instance, in a study conducted by Glyn et al., only 22% of radiation-induced ulcers were amenable to primary closure, with the majority requiring a more complex flap or skin graft reconstruction. These findings emphasize the need for versatile and effective reconstructive options (12).

In this context, the vertical muscle-sparing LD flap has emerged as a valuable technique. Its advantages over the traditional methods are corroborated by the cases discussed herein. This approach not only minimizes surgical time and the need for intraoperative positional changes but also facilitates the preservation of muscle function, particularly by conserving the horizontal branch of the thoracodorsal nerve.

Surgeons performing operations in the operating room must meticulously plan and perform patient positioning to minimize the risk of infection. The surgical team, in collaboration with the surgeon, need to adhere to specific protocols and guidelines to guarantee optimal patient placement, ensuring that a sterile environment is maintained throughout the surgery, thereby decreasing the chances of postoperative infections. Additionally, ensuring correct positioning is essential to preserve asepsis and reduce the likelihood of infection at the surgical site. The placement of a patient on the operating table can affect the visibility of the surgical area, efficiency of sterile draping, and prevention of contamination. Therefore, completing the surgery without changing the surgical position during surgery can be considered one of the most important benefits.

The inclusion of muscles in flap construction, as demonstrated in our cases, has shown superior adhesion and functional outcomes compared with the use of skin flaps alone. This aligns with observations from other studies indicating that muscle flaps generally yield better results than skin flaps in similar reconstructive scenarios (3). Furthermore, the capability of elevating flaps up to 10 cm in width, as observed in our cases, provides ample coverage for substantial defects while maintaining aesthetic integrity. Lee et al. also contributed to this discourse by illustrating the efficacy of immediate reconstruction using a vertical muscle-sparing LD flap after mastectomy (7). Their observations regarding the reduced need for additional draping and the feasibility of operating in a single position further validated the practical benefits of this technique.

In conclusion, the vertical muscle-sparing LD flap represents a significant advancement in the reconstruction of radiation-induced ulcers after breast cancer treatment. Its ability to balance effective coverage with functional preservation makes it an invaluable tool in the reconstructive arsenal, especially in cases complicated by extensive radiation damage.

## Data Availability

The original contributions presented in the study are included in the article/Supplementary Material, further inquiries can be directed to the corresponding authors.
